# Antibacterial and Antibiofilm Activities of Chloroindoles Against *Vibrio parahaemolyticus*

**DOI:** 10.3389/fmicb.2021.714371

**Published:** 2021-08-02

**Authors:** Ezhaveni Sathiyamoorthi, Olajide Sunday Faleye, Jin-Hyung Lee, Vinit Raj, Jintae Lee

**Affiliations:** School of Chemical Engineering, Yeungnam University, Gyeongsan, South Korea

**Keywords:** antibiofilm, antimicrobial, chloroindoles, indole, *Vibrio parahaemolyticus*

## Abstract

*Vibrio parahaemolyticus* is a food-borne pathogen recognized as the prominent cause of seafood-borne gastroenteritis globally, necessitating novel therapeutic strategies. This study examined the antimicrobial and antivirulence properties of indole and 16 halogenated indoles on *V. parahaemolyticus*. Among them, 4-chloroindole, 7-chloroindole, 4-iodoindole, and 7-iodoindole effectively inhibited planktonic cell growth, biofilm formation, bacterial motility, fimbrial activity, hydrophobicity, protease activity, and indole production. Specifically, 4-chloroindole at 20 μg/mL inhibited more than 80% of biofilm formation with a minimum inhibitory concentration (MIC) of 50 μg/mL against *V. parahaemolyticus* and *Vibrio harveyi*. In contrast, 7-chloroindole inhibited biofilm formation without affecting planktonic cell growth with a MIC of 200 μg/mL. Both chlorinated indoles caused visible damage to the cell membrane, and 4-chloroindole at 100 μg/mL had a bactericidal effect on *V. parahaemolyticus* within 30 min treatment, which is superior to the effect of tetracycline at the same dose. The quantitative structure-activity relationship (QSAR) analyses revealed that chloro and bromo at positions 4 or 5 of the indole are essential for eradicating the growth of *V. parahaemolyticus*. These results suggest that halogenated indoles have potential use in antimicrobial and antivirulence strategies against *Vibrio* species.

## Introduction

*Vibrio parahaemolyticus* is a Gram-negative, free-living halophilic bacterium mostly found in estuarine, marine, and coastal environments. The bacterium is a widely known cause of seafood-related gastroenteritis (Lee et al., 2019). Previous studies reported the occurrence of antibiotic-resistant *V. parahaemolyticus* (Cabello et al., 2013; Ashrafudoulla et al., 2019). The adaptation of *Vibrio* species to different aquatic environments and diverse seafood hosts is essential to their survival and colonization (Nithya and Pandian, 2010). Furthermore, *V. parahaemolyticus* strains possess various virulence factors that include biofilm formation, thermostable hemolysin, adhesins, type III and VI secretion systems, extracellular proteases, motility, and fimbriae activity (Salomon et al., 2013; Letchumanan et al., 2014). Both clinical and environmental isolates of *V. parahaemolyticus* showed strong biofilm-forming ability, which increases their cell attachment capacity and resistance to disinfectants and antibiotics (Song et al., 2017; Ashrafudoulla et al., 2019; Lu et al., 2021). The growing emergence of antibiotic resistance due to biofilm formation is a serious public health concern. Therefore, identifying new antimicrobial treatments is urgently needed.

Many Gram-positive and Gram-negative bacteria, including *V. parahaemolyticus*, encode the tryptophanase (*tnaA*) gene in their chromosome and produce indole (Lee and Lee, 2010). Indole and its aromatic heterocyclic structure are popularly used as synthetic starting points in the pharmaceutical industry. Moreover, indole acts as an interspecies and interkingdom signaling molecule that directs various bacterial functions (Lee and Lee, 2010; Lee et al., 2012b, 2015; Kumar et al., 2021). Indole and its derivatives are viewed as potential antivirulence compounds against antibiotic-resistant pathogens because of their ability to inhibit quorum sensing and the production of virulence factors. Specifically, 7-fluoroindole reduced the virulence, hemolysis, protease activity, and biofilm formation of *Pseudomonas aeruginosa* (Lee et al., 2012a). In addition, 5-iodoindole eradicated persister and biofilm formation by *Escherichia coli* and *Staphylococcus aureus* (Lee et al., 2016). Recently, the antimicrobial and antibiofilm activities of halogenated indoles were reported in *Serratia marcescens* and *Acinetobacter baumanni* (Raorane et al., 2020; Sethupathy et al., 2020). Although, indole acts as an extracellular cue regulating gene expression and inhibiting ToxR regulon expression from modulating the virulence gene expression and biofilm formation in *Vibrio cholerae* (Mueller et al., 2009; Howard et al., 2019), the biological activities of indole and its derivatives against *V. parahaemolyticus* needs to be investigated.

This study assessed 16 halogenated indoles as potential potent antimicrobial and antibiofilm agents against *V. parahaemolyticus* and *Vibrio harveyi*. *In vitro* and computational approaches were used to achieve this aim, including cell growth, biofilm, motility, fimbriae, hydrophobicity, protease, time-killing, indole production assays, and quantitative structure-activity relationship (QSAR).

## Materials and Methods

### Strain, Chemicals, and Culture Materials

*V. parahaemolyticus* strain ATCC 17802 and *V. harveyi* ATCC 14126 (American Type Culture Collection, Manassas, United States) were used in this study. Marine Luria-Bertani (mLB) broth containing 3% (w/v) NaCl was purchased from Becton Dickinson (Franklin Lakes, NJ, United States). *V. parahaemolyticus* was streaked from a glycerol stock −80°C onto mLB agar plates and incubated overnight at 30°C. For biofilm and other assays, a single colony from each plate was inoculated into 2 mL of an mLB broth and incubated overnight at 30°C in a shaking incubator at 250 rpm (Ashrafudoulla et al., 2020). Sixteen halogenated indole derivatives (4-bromoindole, 5-bromoindole, 6-bromoindole, 7-bromoindole, 4-chloroindole, 5-chloroindole, 6-chloroindole, 7-chloroindole, 4-fluoroindole, 5-fluoroindole, 6-fluoroindole, 7-fluoroindole, 4-iodoindole, 5-iodoindole, 6-iodoindole, 7-iodoindole, and indole) and other chemicals were purchased from Sigma-Aldrich (St. Louis, MO, United States) and Combi-Blocks, Inc. (San Diego, CA, United States). The compounds were dissolved in dimethyl sulfoxide (DMSO). Kanamycin, tetracycline, and crystal violet were obtained from Sigma-Aldrich. Dimethyl sulfoxide (DMSO) was also used as a negative control in all experiments at a concentration of 0.1% (v/v) in media; it did not affect cell growth or the biofilm formation.

### Biofilm Inhibition Assay

The effect of indole and its derivatives on the biofilm formation by *V. parahaemolyticus* and *V. harveyi* were examined using an assay in 96-well polystyrene plates (SPL life science, Pocheon, South Korea), as previously reported (Rajalaxmi et al., 2016). The bacterial cells were grown in mLB and treated with or without indole and its halogenated derivatives for 24 h at 30°C without shaking. The edge effects were avoided by adding the same amount of mLB to the peripheral wells (300 μL). After incubation, the planktonic cell densities were measured at 620 nm, and the culture supernatant was discarded. The residual planktonic cells were removed by washing the plates three times with water. Then, the biofilms formed in the plates were stained with crystal violet 0.1% for 20 min. The excess dye was removed by washing three times, and the bound crystal violet was solubilized in 95% ethanol (Khadke et al., 2019; Lu et al., 2021). The absorbance was measured at 570 nm using a Multiskan EX microplate photometer (Thermo Fisher Scientific, Waltham, MA, United States).

### Cell Growth and Determination of Minimum Inhibitory Concentrations

For cell growth, the optical densities were measured periodically at 620 nm for 24 h using a Multiskan EX microplate photometer. The minimum inhibitory concentration (MIC) of indole and its halogenated derivatives were determined using broth micro-dilution techniques according to the CLSI (Clinical and Laboratory Standards Institute) with Muller–Hinton Broth containing 3% NaCl in 96-well polystyrene plates. *V. parahaemolyticus* and *V. harveyi* were inoculated into the broth at a dilution of 1:100, dispensed into the peripheral wells (300 μL), and incubated for 24 h with or without the compounds. The MIC for antimicrobial activity is defined as the lowest concentration that inhibits visible cell growth after 24 h culture (Lu et al., 2021). The experiment was performed using at least three independent cultures.

### Swimming and Swarming Motility Assays

For the swimming assay (single movement of bacterial flagella in an aqueous environment), an overnight grown culture (1 μL) of *V. parahaemolyticus* was spotted on the center of mLB plate containing 0.3% agar with or without indole. The plates were kept in the upright position at 30°C for 24 h. The images were photographed, and the radial diameter was measured. Swarming plates were freshly prepared mLB plates containing 0.6% agarose. The plates were dried for 4 h before the experiment, and 1 μL of bacterial culture was spotted in the center. The plates were sealed with plastic tape to accumulate volatile acids and kept in the inverted position at 30°C for 24 h (Heering et al., 2017). The freshly prepared media were used for the motility assay, and the experiments were carried out in triplicate.

### Fimbria Activity Assay

The effects of indole and halogenated indoles on the *V. parahaemolyticus* fimbria activity were accessed using *Saccharomyces cerevisiae* (Sigma-Aldrich, product no. YSC2), as reported previously (Sethupathy et al., 2020). Yeast agglutination was measured spectrophotometrically by adding 1.5 mL of PBS containing 0.5 mL of *S. cerevisiae* (2% w/v in PBS) and 0.4 mL of *V. parahaemolyticus* cells in PBS (OD_600_, 0.5). A uniform mixture of *S. cerevisiae* and *V. parahaemolyticus* cell suspension was achieved by gently vortexing the reaction tubes for 5 s. The initial OD_600_ was measured. After 10 min of incubation at room temperature, 100 μL of the upper phase was transferred to a 96 well plate, and the OD_600_ was measured. The presence of visible aggregates of agglutinated cells affected the OD_600_ measurement. Hence, vigorous vortexing for 30 s was done to disturb the agglutinated cells before reading the second OD_600_ values. The percentage agglutination was calculated using the formula: $100\times \left(\frac{1-{\text{OD}}_{600}\mathrm{before}\mathrm{vortexing}}{{\text{OD}}_{600}\mathrm{after}\mathrm{vortexing}}\right)$.

### Cell Surface Hydrophobicity Assay

The bacterial adherence to hydrocarbons (BATH) test was determined using xylene according to the MATH test method (Mizan et al., 2016). Xylene was used because it possesses greater hydrophobicity than the other hydrocarbons (Ashrafudoulla et al., 2020). *V. parahaemolyticus* was grown in mLB broth at 30°C for 24 h at 250 rpm with halogenated indoles. The cells were harvested by centrifugation at 11,000 × g for 15 min. The pellets were washed twice with a PBS (pH 7.2). The cell density was adjusted to 0.5 at 600 nm (A_0_), and 4 mL of cell suspension was transferred to individual glass tubes containing 1 mL of xylene. The mixture was vortexed vigorously for 1 min and left on the bench for 30 min at room temperature to allow the two phases to separate. The aqueous phase was then removed carefully to measure the OD_600_ of the cells remaining in suspension (A_*i*_). The assays were performed in triplicate. The mean percentage adherence to xylene hydrophobicity was estimated using the following formula:

$\text{Hydrophobicity}\left(\text{H}\right)\%=\frac{\left({\text{A}}_{\text{o}}-{\text{A}}_{\text{i}}\right)}{{\text{A}}_{\text{o}}}\times 100$ and strains were classified into three categories: not hydrophobic (<20%), moderate (20–50%), and strong (>50%).

### Protease Assay

The extracellular casein-degrading protease activities of the supernatant from *V. parahaemolyticus* grown in the presence or absence of indole compounds were measured using 2% w/v of azocasein (Sethupathy et al., 2020). The culture was diluted (1:100) in fresh mLB supplemented with various concentrations of halogenated indoles, kept at 24 h with 250 rpm at 30°C, and centrifuged at 15,000 × g for 15 min. The culture supernatant (75 μL) was mixed with 125 μL of azocasein and kept at 37°C for 30 min. Subsequently, 600 μL of 10% trichloroacetic acid was added to stop the proteolysis. The reaction tubes were kept at −20°C for 30 min to precipitate the unreacted azocasein and the precipitates were removed by centrifugation at 10,000 × g for 10 min. A 600 μL sample of supernatant and 700 μL of NaOH were added, and the absorbance was read at 440 nm. The experiment was carried out in triplicate.

### Indole Kovac’s Assay

The effect of halogenated indoles on indole production was measured using Kovac’s reagents (Darkoh et al., 2015). Briefly, *V. parahaemolyticus* was treated with or without indole compounds at 30°C with shaking at 250 rpm for 10 h. The grown culture (1 mL) was removed and centrifuged at 11,300 × g for 5 min. The supernatant was removed, and 300 μL of Kovacs reagent (10 g of *p*-dimethyl amino benzaldehyde dissolved in 50 mL of HCl and 150 mL of amyl alcohol) was added. The mixture was incubated for 2 min, and 50 μL was taken and added to 1 mL of a HCl-amyl alcohol solution (mixture of 75 mL of HCl and 225 mL of amyl alcohol). The absorbance was measured at 540 nm. Additionally, planktonic cell growth in relation to extracellular indole production by *V. parahaemolyticus* was determined in mLB broth at 30°C with shaking at 250 rpm for 24 h.

### Rapid-Killing Assay

To determine the rapid-killing efficacy of indoles, overnight cultures of *V. parahaemolyticus* were treated with the antibiotics (kanamycin and tetracycline), 4-choroindole, 7-chloroindole, 4-iodoindole, or 7-iodoindole, and incubated for 0, 15, 30, and 60 min at 30°C with shaking at 250 rpm. At the indicated time points, a 100 μL aliquot of the treated cells was harvested, diluted with PBS as required, and plated on mLB agar plates. After incubation at 30°C for 24 h, the colony-forming units (CFUs) were counted (Sampson et al., 2012; Raorane et al., 2020). Compared to the initial CFU, the decrease in CFU by the addition of indoles or antibiotics indicates killing capacity of tested compounds. While MIC indicates antimicrobial activity, this rapid-killing assay is used to study the rapid killing capacity of compounds. The experiment was repeated three times independently; the mean and standard deviation vs. times were plotted on a logarithmic graph.

### Confocal Laser Scanning Microscope for Biofilm Observation

*V. parahaemolyticus* was inoculated in 96-well polystyrene plates (SPL life science, South Korea) without shaking at 30°C for 24 h with or without the indoles. The biofilms were stained with 100 μL of pre-warmed PBS containing CFSE stain (carboxyfluorescein diacetate succinimidyl ester) (Invitrogen, Molecular Probes, Inc., Eugene, OR, United States) for 20 min at 37°C (final concentration, 5 μM) and washed three times with PBS. The static biofilm plates were visualized by excitation using Ar 488 nm (emission wavelengths 200–550 nm). The cells were visualized by confocal laser microscopy (Nikon Eclipse Ti, Tokyo, Japan) using a 20× objective. DMSO was used as the control. Color confocal images were visualized using NIS-Elements C version 3.2 (Nikon eclipse). For each experiment, at least 10 random positions in three independent cultures were chosen for microscopic analysis (Sethupathy et al., 2020).

### Scanning Electron Microscopy for the Cell Morphology

The cell morphology of *V. parahaemolyticus* was investigated by SEM (S-4100, Hitachi, Tokyo, Japan). The cells were grown in 250 mL glass conical flasks containing 25 mL of mLB for 1 h in the presence of 4-chloroindole, 7-chloroindole, and tetracycline at 200 μg/mL. The culture was then filtered through a nylon membrane filter (0.5 cm × 0.5 cm) using a 0.45 μm nylon filter under vacuum. The cells were fixed with 2.5% glutaraldehyde and 2% formaldehyde for 24 h, post-fixed in PBS and osmium tetroxide, and dehydrated using a graded series of ethanol (50, 70, 80, 90, 95 and 100%; 15 min each), and isoamyl acetate. After critical-point drying, the cells on filters were sputter-coated with palladium/gold and observed by SEM at magnifications ranging from ×1,000 to 10,000 at an accelerating voltage of 15 kV.

### Generation of 3D-QSAR Models of Halogenated Indoles Against *V. parahaemolyticus*

The Schrödinger suite (Maestro 11.4, New York, United States) was used to predict the 3D-QSAR of halogenated indoles. The 2-D structures of these halogenated indoles were drawn in ChemDraw Ultra version 12 (CambridgeSoft), and their geometries were optimized using GaussView 6.0. The structures were then saved as SDF files. Later, the MIC values of the indoles against *V. parahaemolyticus* were converted to the corresponding pMIC [-log (MIC)] values and used for the QSAR calculations. In this study, an atom-based 3D-QSAR model was employed for each of the 16 halogenated indoles. For the development of QSAR, Van der Waals’s models of aligned training sets of molecules were located in a standard grid of cubes. The 12 halogenated indoles in the training set and five halogenated indoles in the test set were assigned to generate the 3D-QSAR proposed hypothesis. Subsequently, a one-component partial least square (PLS) factor model was used.

### Statistical Analysis

All experiments were performed with two independent cultures and six repetitions. The data are presented as the mean values ± SD. The differences between means were tested using a student *t*-test. Differences were considered significant at *p* = 0.05.

## Results

### Antimicrobial and Antibiofilm Activities of Halogenated Indoles Against *V. parahaemolyticus* and *V. harveyi*

**FIGURE 1:**
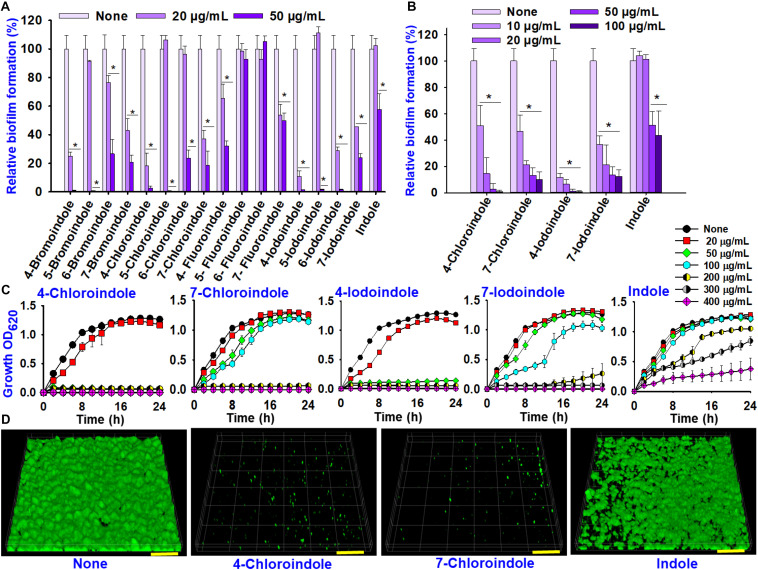
Effects of indole and halogenated indoles on biofilm formation and planktonic cell growth against *V. parahaemolyticus*. The antibiofilm activity of halogenated indoles **(A)**. Antibiofilm activity of four selected halogenated indoles and indole **(B)**. Growth curve of *V. parahaemolyticus* treated with different concentration of the four selected halogenated indoles and indole **(C)**. CLSM images of *V. parahaemolyticus* biofilms with or without 50 μg/mL of 4-chloroindole, 7-chloroindole, and indole **(D)**. The scale bar is 100 μm. Asterisks (*) represent significant differences (*p* < 0.05).

In order to identify compounds with antimicrobial and antibiofilm properties, indole and 16 halogenated indoles at 20 and 50 μg/mL were initially assessed against *V. parahaemolyticus* for their ability to inhibit planktonic cell growth and biofilm formation. Overall, the halogenated indoles demonstrated a wide range of inhibitory activities on the biofilm formation of *V. parahaemolyticus* (Figure 1A). Although most of the indoles showed marked antibiofilm activity, 5-fluoroindole, 6-fluoroindole, and indole showed less appreciable activity. Six halogenated indoles (4-bromoindole, 7-bromoindole, 4-chloroindole, 7-chloroindole, 4-iodoindole and 7-iodoindole) at 20 μg/mL reduced biofilm formation significantly (by more than 50%) in *V. parahaemolyticus*. Of the hits, four halogenated indoles (4-chloroindole, 7-chloroindole, 4-iodoindole, and 7-iodoindole) with higher activities were selected for further investigation.

4-Bromoindole and 7-bromoindole were excluded because bromo compounds often showed cell cytotoxicity (Kammann et al., 2006). Hence, greater focus was placed on the potential of chlorinated indoles in this study. The selected halogenated indoles were tested further at various concentrations (10, 20, 50, and 100 μg/mL) (Figure 1B). 4-Chloroindole and 4-iodoindole exhibited the strongest antibiofilm activity because they inhibited planktonic cell growth in addition to biofilm formation (Figures 1B,C). On the other hand, 7-chloroindole and 7-iodoindole showed antibiofilm ability with weak inhibitory activity on planktonic cell growth.

Interestingly, the backbone indole exhibited less antibacterial and antibiofilm activity, while indole at 50 μg/mL decreased biofilm formation by approximately 50% without affecting its planktonic cell growth (Figure 1B). The current *V. parahaemolyticus* produced approximately 55 μg/mL indole in the stationary growth phase (Supplementary Figure 1). This is the first report showing that indole inhibits its own biofilm formation in *V. parahaemolyticus*. This observation supports that indole is an important biofilm modulator and bacterial signal (Lee and Lee, 2010).

CLSM analysis was used to confirm the antibiofilm properties of 4-chloroindole and 7-chloroindole. While *V. parahaemolyticus* ATCC 17802 used in this study formed strong biofilm as shown in the control, 4-chloroindole and 7-chloroindole drastically reduced the surface coverage and biofilm biomass (Figure 1D). On the other hand, indole at the same concentration had a minor effect on the biofilm characteristics. The MIC assay was carried out to examine the antibacterial profiles of the halogenated indoles (Table 1). Among the 16 halogenated indoles, 4-bromoindole, 5-bromoindole, 4-chloroindole, and 5-chloroindole showed the lowest MIC (50 μg/mL) while indole had the highest MIC (400 μg/mL). Furthermore, other antibiofilm compounds, 7-chloroindole and 7-iodoindole, exhibited moderate antibacterial activity with MICs of 200 and 275 μg/mL, respectively.

**TABLE 1:**
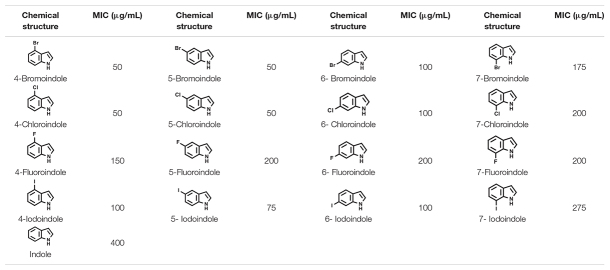
Minimum inhibitory concentration (MICs) of the halogenated indoles in *V*. *parahaemolyticus*.

Additionally, antimicrobial and antibiofilm activities of indoles were investigated against another *vibrio* strain, *V. harveyi* ATCC 14126. The trend of antimicrobial and antibiofilm efficacies of halogenated indoles against *V. harveyi* was very similar to that of *V. parahaemolyticus* (Supplementary Table 1 and Supplementary Figure 2). These results indicate the broad-spectrum efficacy of halogenated indoles against *Vibrio* spp.

### Halogenated Indoles Inhibited Motility and Fimbriae Activity

**FIGURE 2:**
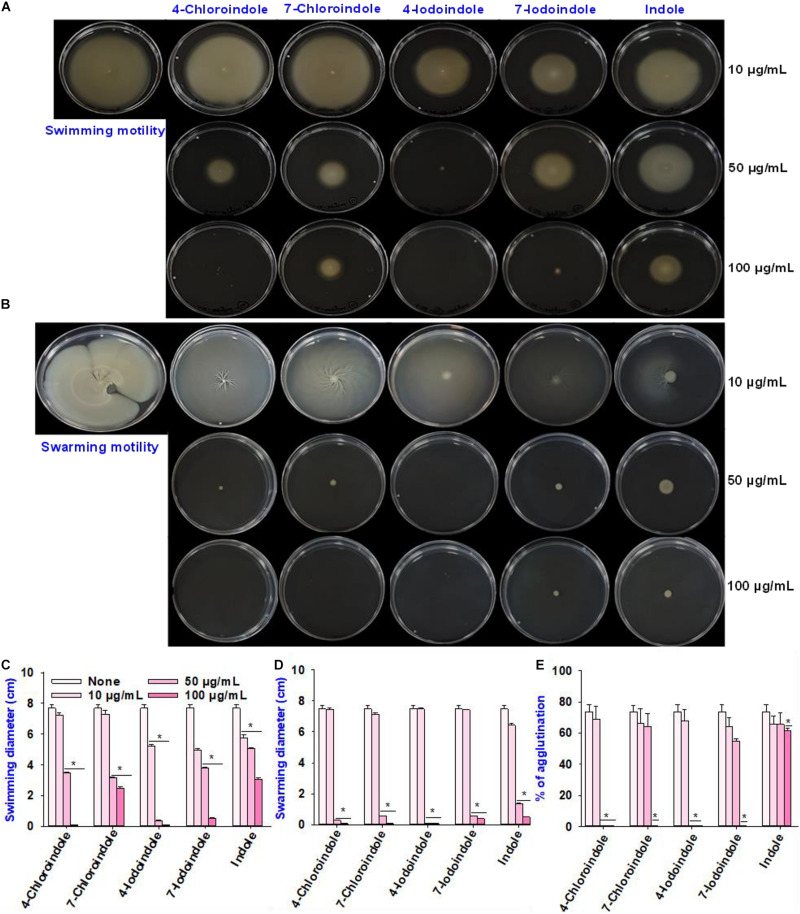
Effects of halogenated indoles on the motility of *V. parahaemolyticus.* Swimming motility **(A)**, swarming motility **(B)**, measurement of migration in the swimming assay **(C)**, measurement of migration in the swarming assay **(D)**, and fimbria-mediated yeast agglutination **(E)**. Asterisks (*) represent significant differences (*p* < 0.05).

In *V. parahaemolyticus*, flagella-mediated motility plays an important role in its pathogenesis and promotes the initial stages of biofilm formation in the human intestine (Kassen and Rainey, 2004). Hence, the effects of the selected halogenated indoles and indole on the swimming and swarming motilities of *V. parahaemolyticus* were investigated (Figure 2). All indoles tested reduced the swimming motility at 100 μg/mL, while 4-chloroindole, 7-chloroindole, 4-iodoindole, and 7-iodoindole at 50 μg/mL decreased the motility more appreciably than indole (Figure 2A). Similarly, indole and the four halogenated indoles almost abolished the swarming motility of *V. parahaemolyticus* even at 50 μg/mL (Figure 2B). On the other hand, at 10 μg/mL, both indole and its halogenated derivatives did not show significant effects on the swimming and swarming motilities of *V. parahaemolyticus* as quantified by the diameters of the swimming and swarming areas (Figures 2C,D).

For successful biofilm formation in *V. parahaemolyticus*, the bacterial surface pili are required for colonization and enable them to attack the host and evade the immune responses by modulating the expression of numerous types of type IV pili (Shime-Hattori et al., 2006; Aagesen and Häse, 2012). Hence, this study evaluated the effects of indole and four halogenated indoles on fimbria-mediated yeast agglutination. The result showed that the four halogenated indoles reduced fimbriae production while backbone indole had negligible effect (Figure 2E). Although 4-chloroindole and 4-iodoindole abolished fimbria production at 50 μg/mL, 7-chloroindole and 7-iodoindole eliminated it at 100 μg/mL. This observation suggests that the biofilm inhibitory activity might be due partly to the ability of the halogenated indoles to interfere with pili and fimbriae production.

### Effects of Halogenated Indoles on the Hydrophobicity, Protease Activity, and Indole Production

**FIGURE 3:**
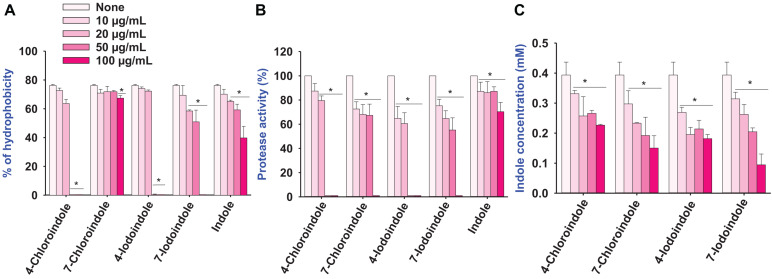
Effect of halogenated indoles on the virulence factors of hydrophobicity **(A)**, protease activity **(B)**, and indole concentration **(C)** of *V. parahaemolyticus.* The error bars and asterisks (*) represent standard deviations and significant differences (*p* < 0.05), respectively, vs. the non-treated controls.

The cell surface hydrophobicity plays an important role in bacterial attachment to a biotic or abiotic surface and biofilm formation (Mizan et al., 2016), the change in cell hydrophobicity by halogenated indoles was also investigated (Figure 3A). Compared to the untreated control, 4-chloroindole and 4-iodoindole strongly reduced the hydrophobicity at 50 μg/mL. In contrast, 7-chloroindole and 7-iodoindole showed minor change and indole showed negligible change at the same concentration.

Protease secretion also plays a critical role in establishing an infection in the host and subsequent breakdown of the host defense by degrading proteins (Duarte et al., 2016). The pathogenesis of *V. parahaemolyticus* is associated with exo-protease production (Lee et al., 2018). Compared to the untreated control, the halogenated indoles reduced exo-protease activity significantly in a dose-dependent manner (Figure 3B). In particular, 4-chloroindole and 4-iodoindole at 50 μg/mL markedly reduced the protease activity, while 7-chloroindole and 7-iodoindole had a moderate inhibitory effect.

Because *V. parahaemolyticus* produces the signaling molecule indole and indole inhibited ToxR regulon expression from modulating virulence gene expression and biofilm formation by *V. cholerae* (Howard et al., 2019), the effect of halogenated indoles on indole production was investigated. Interestingly, four of the halogenated indoles tested exhibited dose-dependent inhibitory effects on indole production in *V. parahaemolyticus* (Figure 3C). It is notable that halogenated indoles can suppress indole production as indole derivatives interfere with indole signaling in *V. parahaemolyticus.*

### Rapid Killing by 4-Chloroindole and 4-Iodoindole

**FIGURE 4:**
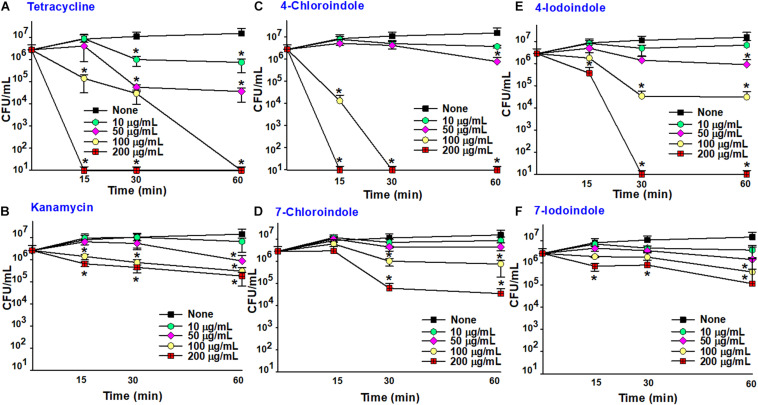
Time-killing assay against *V. parahaemolyticus.* Tetracycline **(A)**, kanamycin **(B)**, 4-chloroindole **(C)** 7-chloroindole **(D)**, 4-iodoindole **(E)**, and 7-iodoindole **(F).** Asterisks (*) represent significant differences (*p* < 0.05).

The fast-killing assay was performed over a short period, and the efficacy of halogenated indoles was compared with that of commercial antibiotics (Figure 4). As expected, bactericidal tetracycline killed the bacterium rapidly (Figure 4A) while another bactericidal, kanamycin, killed it slowly (Figure 4B). Interestingly, 4-chloroindole and 4-iodoindole exhibited rapid bactericidal effects, while 7-chloroindole and 7-iodoindole showed slow killing. For example, 4-chloroindole at 100 μg/mL reduced cell survival by 2-log10 of CFU within 15 min and by more than 5-log10 of CFU after 30 min (Figure 4C). Its rapid killing efficacy is superior to that of tetracycline at the same concentration (Figure 4A). Also, 4-iodoindole at 200 μg/mL exerted rapid bactericidal activity (Figure 4E). Previous study reported that rapid antibacterial activities of antibiotics began immediately after they were introduced (Foerster et al., 2016). Also, shorter duration of antibiotic administration can reduce adverse effects associated with their use.

### Cell Morphological Change by Halogenated Indoles

**FIGURE 5:**
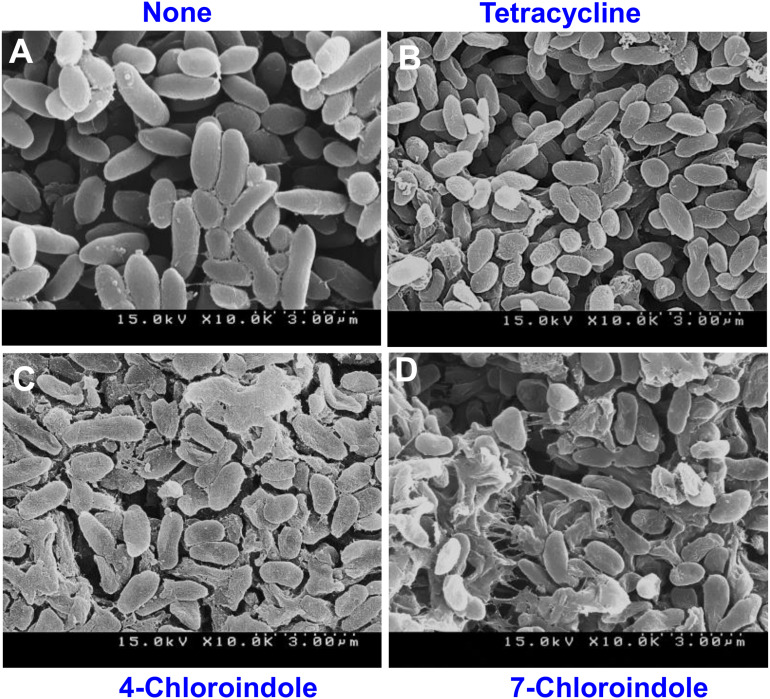
SEM images of *V. parahaemolyticus* cultured with indoles and tetracycline at 200 μg/mL. None **(A)**, tetracycline **(B)**, 4-chloroindole **(C)**, and 7-chloroindole **(D)**.

The morphological changes in *V. parahaemolyticus* cells treated with 4-chloroindole, 7-chloroindole, and tetracycline were observed by SEM. Normal *V. parahaemolyticus* cells were typically rod-shaped (Figure 5A) while treatment with tetracycline (Figure 5B) showed abnormal texture and smaller coccoid forms, as reported previously (Wang et al., 2013). Interestingly, the cells treated with 4-chloroindole and 7-chloroindole were changed significantly with membrane shrinkage, showing the formation of pits in the cell envelope, membrane rupture, and irregular cell shape (Figures 5C,D).

### Deciphering QSAR of Halogenated Indoles With Their Antibacterial Activity Against *V. parahaemolyticus*

**FIGURE 6:**
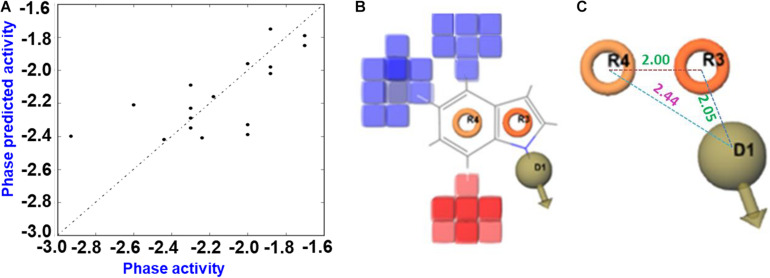
QSAR prediction of halogenated indoles against *V. parahaemolyticus*. Fitness diagram between the observed and phase-predicted activity halogenated indoles **(A)**, 3D-QSAR representation of various substituents (blue cubes with positive potential and red cubes with negative potential) **(B)**, and essential pharmacophore halogenated indoles against *V. parahaemolyticus* **(C)**.

The generated 3D-QSAR model was used to decipher the SAR between the halogenated indoles and their antibacterial activities (Figures 6A–C). Halogenated indoles having a MIC of >200 μg/mL were used as test sets in the experiments, and the remaining indoles were assigned to the training sets. Initially, the pharmacophore hypothesis was generated using these train and test sets, where the developed pharmacophore pattern showed a statistically considerable range of ligand fitness 2.91–3.0 with a PLS regression factor of one and three matched sites of halogenated indoles (Supplementary Table 2). Furthermore, to predict the atom-based 3D-QSAR model, D; Hydrogen donor, R; aromatic rings (DRR) pharmacophore patterns were selected to find a higher degree of robustness (70.3) (Supplementary Table 3). Furthermore, the quality of the QSAR model parameters, such as the standard deviation (SD), cross-validated Pearson’s coefficient (r^2^), and degree of freedom (F), were found to be in the range of 0.086, 0.88, and 70.3, respectively. These statistical ranges of parameters represent the considerable range of linearity and quality of the predicted 3D-QSAR model. Moreover, the lower variance ratio (p) and higher F-value also indicated the statistical significance of the predicted QSAR model (Supplementary Table 3). The R2 value and root mean square error reflects the predictability, while a lower SD value showed the best fitness of the QSAR model. The data values plotted around the best-fit lines provide the significance of the creative model. The similarity between the trends of the predicted activity and actual activity showed good forecasting of the model. Based on these static parameters, the predicted 3D-QSAR showed the accuracy of the cube color and the predicted reliable QSAR model of indoles. The 3D structural feature of the QSAR model provided the contour cubes on the favorable and unfavorable regions on the various atomic positions of halogenated indoles (Figure 6B). Based on the coefficient values for the aromatic ring and hydrogen donor with a negative coefficient = -1.200e-001 and a positive coefficient = 1.200e-001, the colors were displayed as the 3D effect of the atomic cubes of halogenated indoles (Figure 6B). The dark blue color showed a positive coefficient, i.e., increased activity, while the red color for the negative coefficient is an indicator of decreased activity. Consequently, the pattern of arrangement of aromatic ring R4 and R3 and the hydrogen donor D1 (Figure 6C) and 3D-QSAR demonstrated that the color cube of the indole ring is essential for the antibacterial activity of halogenated indoles against the assigned bacterial strain. The antibacterial activity increased by the governing blue color at the C4 and C5 positions (bromoindoles and chloroindoles) of the indole motif owing to higher electronegative atoms, such as chloro and bromo. On the other hand, the 7th position (red color) did not participate significantly in the antibacterial activity against *V. parahaemolyticus*. In contrast, the substitution of a halogen at the position of C6 exhibited moderate antibacterial activity. Overall, these observations suggested that nucleophilic substitution, such as -Cl and -Br at the 4th position and 5th position (blue color) of indoles, contributes to the increase in antibacterial activity against *V. parahaemolyticus*.

## Discussion

This is the first report assessing the biological activity of indole and its derivatives against *V. parahaemolyticus* and *V. harveyi.* Indole and its derivatives have been investigated for their antivirulence activity against biofilm-forming pathogens, such as *P. aeruginos*a, *E. coli*, *S. marcescens*, and *S. aureus* (Lee et al., 2007, 2013; Lee and Lee, 2010; Sethupathy et al., 2020). Indole is produced by more than 85 bacterial species and is considered an intercellular signaling molecule exploited by microbial communities (Lee and Lee, 2010). The functional groups of the indole derivatives control the significant role in several virulence factors of *V. parahaemolyticus* (Figures 1, 2). This is the first report showing that indole inhibits its own biofilm formation in *V. parahaemolyticus*. This observation supports that indole is an important biofilm modulator and bacterial signal (Lee and Lee, 2010).

The biofilm-forming *Vibrio* species are a major threat to the aquaculture industry (Nithya and Pandian, 2010). Because biofilm cells are entrapped within extracellular polymeric substance (EPS), it is desirable to have antimicrobial agents that can penetrate the biofilms, kill the bacterial cells, decrease cell adhesion, and inhibit the formation of EPS/biofilms. The results of this study suggest that 4-chloroindole and 7-chloroindole have a reasonable biofilm inhibitory effect with MICs of 50 and 200 μg/mL, respectively (Figure 1). The lower MIC observed for 4-chloroindole and 4-iodoindole explains why they inhibited both biofilm formation and cell growth significantly. A previous study reported that 6-chloroindole and 6-chlorooxindole had antibacterial activities against marine bacteria—*Bacillus subtilis* and *P. aeruginosa* (Yang et al., 2014). On the other hand, the antibiofilm activities of 7-chloroindole and 7-iodoindole with higher MICs highlight their potential as an antivirulence agent against *V. parahaemolyticus* because they exhibit more inhibitory effects on biofilm formation than the free-growing cells. This suggests that the tested bacterium will have less tendency to develop resistance against them because they did not disrupt their planktonic cells. Based on the MIC values obtained, the antimicrobial activities of these compounds were in this order: 4-bromoindole, 5-bromoindole, 4-chloroindole, 5-chloroindole > 5-iodoindole > 6-bromoindole, 6-chloroindole, 4-iodoindole, 6-iodoindole > 4-fluoroindole > 7 bromoindole > 7-chloroindole, 5-fluoroindole, 6-fluoroindole, 7-fluoroindole > 7-iodoindole > indole (Table 1). Also, the ranking of antibiofilm activities were in this order: 4-bromoindole, 5-bromoindole, 5-chloroindole > 4-iodoindole, 5-iodoindole, 6-iodoindole, 4-chloroindole > 7-chloroindole > 7-bromoindole, 6-chloroindole, 6-bromoindole, 7-iodoindole > 4-fluoroindole > 7-fluoroindole > indole > 5-fluoroindole > 6-fluoroindole at 50 μg/mL (Figure 1A).

Cell motility, such as swimming and swarming ability, helps the cells colonize the host or abiotic surfaces (Verstraeten et al., 2008). In particular, four selected halogenated indoles reduced the swimming and swarming motilities of *V. parahaemolyticus* significantly (Figure 2). Swarming motility is associated with quorum sensing and the ability to form biofilms in pathogenic bacteria (Overhage et al., 2008; Lee et al., 2009). Previously, 7-fluoroindole abolished the swarming motility of the *P. aeruginosa* PA14 strain but not the swimming motility (Lee et al., 2012a). Recently, 5-fluoroindole, 6-fluoroindole, 5-methylindole, and 7-methyl indole reduced the swarming and swimming motilities in *S. marcescens* (Sethupathy et al., 2020). And this study reports a direct correlation between the motility and biofilm formation with halogenated indoles in *V. parahaemolyticus* (Figures 1, 2). Similarly, adhesion is a key step in biofilm development, and various fimbrial adhesins have been shown to play a role in biofilm formation in *V. parahaemolyticus* (Shime-Hattori et al., 2006). This suggests that the biofilm inhibitory activity might be due partly to the ability of the halogenated indoles to interfere with fimbriae production.

The halogenated indoles inhibited the cell hydrophobicity and protease activity in a dose-dependent manner (Figures 3A,B). These findings agree with a previous report that fluoroindoles decreased the protease activity of *P. aeruginosa* while indole had negligible effects (Lee et al., 2012a). A previous study reported a good correlation between protease production and biofilm-forming ability as well as quorum signals among the isolates of *V. parahaemolyticus* investigated (Mizan et al., 2016). Hence, the inhibition of protease activity in this study might have contributed to the biofilm inhibitory capacities of the halogenated indoles. Furthermore, the hydrophobicity index is an important virulence factor for cell-cell communication that can vary under different environmental stress and nutrient deficiencies (Barrasso et al., 2021; Park et al., 2021). The observed biofilm reduction and the decrease in hydrophobicity demonstrated by these halogenated indoles corroborate the reports that the biofilm-forming ability of *V. parahaemolyticus* positively correlated with the cell surface hydrophobicity (Mizan et al., 2016).

Interestingly, the four halogenated indoles dose-dependently inhibited indole production in *V. parahaemolyticus* (Figure 3C). Indole plays important roles in various bacterial phenotypes and even in eukaryotic immunity (Lee and Lee, 2010; Lee et al., 2015; Kumar et al., 2021). Particularly, indole has been considered as a possible quorum sensing molecule that modulate biofilm formation and virulence in various bacteria (Lee et al., 2015, 2016). Therefore, the inhibition of indole production by halogenated indoles supports their potential as quorum quenching agents against *Vibrio* species.

The swarming and swimming motilities, protease activity, hydrophobicity, fimbriae, and indole production are associated with biofilm formation and other virulence activities in *V. parahaemolyticus*. Hence, the inhibition of these virulence factors by halogenated indoles supports their antibiofilm potentials.

The consequences of the QSAR model showed that the pharmacophore arrangement DRR is essential for the halogenated indoles series to produce a significant level of antibacterial activity against *V. parahaemolyticus*. Visualization of the cubes in the QSAR model indicates the receptor site topology, effectiveness of the ligand, and non-covalent interactions with the receptor. Nucleophilic substitution in the six-member aromatic ring of indole, particularly at the 4th and 5th position, help enhance the antibacterial activity. These halogenated atoms might be interacting with the electrophilic atoms of the amino acids of the bacterial cellular receptor proteins, which can interfere with the architecture of bacterial membrane development. In contrast, substitution by halogen atoms at the 7th position is not essential compared to the 6th position of indole. QSAR model was developed on the basis of MIC values against *V. parahaemolyticus* to reveal the indoles structure co-relation with their biological activity (MIC against *V. parahaemolyticus*). The MIC was converted into log values and then used in the QSAR model. The activity range as per log MIC value =200 μg/mL was assigned inactive range in QSAR model. Overall, Br and Cl at the 4th and 5th positions of indole, respectively, suggest that 4-chloroindole, 4-bromoindole, 5-chloroindole, and 5-bromoindole (MIC 50 μg/mL) are the lead molecules for the development of antibacterial agents against *V. parahaemolyticus*. This is because they possess better potency (MIC 50 μg/mL) as compared to 7-chloroindole (MIC 200 μg/mL). However, the indole inhibition (Figure 3C) does not follow the QSAR model. Further study will be needed to elucidate the plausible mechanism of action of these halogenated indoles against *V. parahaemolyticus*.

Previously, 5-iodoindole and 7-fluoroindole were most effective antivirulence agents against *A. baumanii* and *P. aeruginosa*, respectively (Lee et al., 2012a; Raorane et al., 2020). In the present study, however, chloroindoles, such as 4-chloroindole and 7-chloroindole, exhibited inhibitory effects on cell growth, biofilm formation, and other virulence factors of *V. parahaemolyticus*, whereas indole had a mild inhibitory effect with a MIC of 400 μg/mL. This suggests that the presence of chlorine atoms in the indole moiety might have been responsible for the antibacterial and antibiofilm activities of the chlorinated derivatives. This claim is supported by a previous study that the presence of a chlorine atom enhanced the antibacterial properties of a chlorine-containing organic compound against *E. coli* (Mujumdar et al., 2018). The presence of chlorine atoms plays a critical role in some natural products, such as the antibiotics clindamycin (Hileman, 1993), vancomycin (Henschler, 1994a; Fang et al., 2019), and chloramphenicol (Henschler, 1994a). The majority of halogenated drugs are fluorinated drugs (57%), followed by chlorinated ones 38% (Hernandes et al., 2010). However, chlorine is a moderate halogen bond acceptor in addition to being larger than fluorine. The C-Cl bond is stable enough, and the subunits bearing it can be accommodated in the hydrophobic pockets of the biological targets (Siegal et al., 2007). Hence, greater focus was placed on the potentials of chlorinated indoles in this study. The mechanism of action of halogenated drugs is unclear. On the other hand, the addition of a chlorine atom into one or more specific positions of a biologically active molecule may improve the intrinsic biological activity substantially (Fang et al., 2019). Previously, the properties of the carbon-chlorine bond (C-Cl) in organochlorines were analyzed (Henschler, 1994a; Henschler and Marquardt, 1995). In the low-molecular-weight chemicals they investigated, however, the electrophilic reactivity of the carbon center adjacent to the chlorine atom, which often causes the displacement of chlorine by (bio) nucleophiles, determines the observed biological properties (Henschler, 1994b). The increased lipophilicity of the whole molecule by a chlorine substituent will lead to a higher partitioning of a chlorinated compound into the lipophilic phase of a cell membrane or the lipophilic domains of a protein. This causes a higher local concentration of the compound near a biological target site and suggests that the chlorinated indoles in the present study might have exhibited antibacterial activities by disrupting the cell membrane of *V. parahaemolyticus*. This assumption is partially supported by the substantial structural damage observed in the cells treated with 4- chloroindole and 7-chloroindole (Figure 5). Furthermore, it was reported that the position of the halogen on the indole moiety plays an essential role in their action against microbial cells (Rajasekharan et al., 2017). The presence of a chlorine atom at the 4th position of the indole ring contributed to bioactivity, as confirmed by the QSAR analysis (Figure 6B).

To the best of the authors’ knowledge, this is the first study to report that halogenated indoles show antibacterial, antibiofilm, and antivirulence activities against *V. parahaemolyticus.* These results suggest that 4-chloroindole and 7-chloroindole can be used as a constituent of biofilm-fighting agents because chlorine-containing disinfectants have been used as broad-spectrum germicides in food processing to limit the presence of pathogenic and spoilage bacteria. In addition, the mechanism of action and the toxicity profile of chloroindoles should be investigated using an *in vivo* model. The QSAR of halogenated indoles helped to understand the halogenated substitution and their impact on the antibacterial activity against *V. parahaemolyticus*, which provides insight into the plausible antibacterial molecular mechanism. Nucleophilic substitution, such as -Cl and -Br at the 4th and 5th positions of indole, is essential to increase the inhibitory potency of the indole scaffold toward *V. parahaemolyticus*. Overall, 4-chloroindole, 4-bromoindole, 5-chloroindole, and 5-bromoindole can be used as lead molecules for discovering alternative antibacterial agents against *V. parahaemolyticus.*

## Data Availability Statement

The original contributions presented in the study are included in the article/Supplementary Material, further inquiries can be directed to the corresponding author/s.

## Author Contributions

J-HL and JL: conceptualization, project administration, and funding acquisition. ES and J-HL: methodology. ES and VR: software. ES, VR, J-HL, and JL: validation. ES, VR, and OF: formal analysis. ES, OF, and J-HL: investigation. JL: resources and supervision. ES, OF, and JL: data curation. ES, J-HL, and JL: writing manuscript. ES, VR, and JL: visualization. All authors have read and agreed to the published version of the manuscript.

## Conflict of Interest

The authors declare that the research was conducted in the absence of any commercial or financial relationships that could be construed as a potential conflict of interest.

## Publisher’s Note

All claims expressed in this article are solely those of the authors and do not necessarily represent those of their affiliated organizations, or those of the publisher, the editors and the reviewers. Any product that may be evaluated in this article, or claim that may be made by its manufacturer, is not guaranteed or endorsed by the publisher.
