# Supplementary material for: Antibacterial and Antibiofilm Activities of Chloroindoles Against Vibrio parahaemolyticus
Source: Front Microbiol. 2021 Aug 2;12:714371. doi: 10.3389/fmicb.2021.714371 (PMC8365150; doi:10.3389/fmicb.2021.714371)
Supplement: Supplementary file 1 [file Data_Sheet_1.DOCX]

**Supplementary information**

**Antibacterial and antibiofilm activities of chloroindoles against *Vibrio parahaemolyticus***

Ezhaveni Sathiyamoorthi^§^, Olajide Sunday Faleye^§^, Jin-Hyung Lee, Vinit Raj,

and Jintae Lee*

School of Chemical Engineering, Yeungnam University, 280 Daehak-Ro, Gyeongsan, 38541,

Republic of Korea

^§^These authors contributed equally to this work.

*Corresponding Author

E-mail: jtlee@ynu.ac.kr. Tel.: +82-53-810-2533. Fax: +82-53-810-4631.

**Supplementary Table 1:** Minimum inhibitory concentrations (MICs) of halogenated indoles against *V*. *parahaemolyticus* and *V. harveyi*.

| Halogenated Indoles | *V.parahaemolyticus*  MIC (µg/ml) | *V.harveyi*  MIC (µg/ml) |
| --- | --- | --- |
| 4-Bromoindole | 50 | 50 |
| 5-Bromoindole | 50 | 50 |
| 6-Bromoindole | 100 | 100 |
| 7-Bromoindole | 175 | 125 |
| 4-Chloroindole | 50 | 50 |
| 5-Chloroindole | 50 | 50 |
| 6-Chloroindole | 100 | 100 |
| 7-Chloroindole | 200 | 175 |
| 4-Fluoroindole | 150 | 125 |
| 5-Fluoroindole | 200 | 175 |
| 6-Fluoroindole | 200 | 200 |
| 7-Fluoroindole | 200 | 250 |
| 4-Iodoindole | 100 | 50 |
| 5-Iodoindole | 75 | 75 |
| 6-Iodoindole | 100 | 75 |
| 7-Iodoindole | 275 | 225 |
| Indole | 400 | 375 |

**Supplementary Table 2.** Fitness and predicted activity data for the test and training set of the halogenated compounds.

| **Ligand name** | **QSAR set** | **Activity** | **PLS factors** | **Predicted Activity** | **Pharm set** | **Fitness** |
| --- | --- | --- | --- | --- | --- | --- |
|  | training | -1.880 | 1 | -1.95 | active | 2.91 |
|  | training | -1.700 | 1 | -1.73 | active | 3.00 |
|  | test | -2.930 | 1 | -2.05 | inactive | 2.91 |
|  | test | -2.240 | 1 | -2.39 | inactive | 2.91 |
|  | training | -1.880 | 1 | -1.97 | active | 2.91 |
|  | training | -1.700 | 1 | -1.77 | active | 2.99 |
|  | training | -2.000 | 1 | -2.05 | active | 2.91 |
|  | training | -2.300 | 1 | -2.31 | inactive | 2.91 |
|  | training | -2.180 | 1 | -2.03 | inactive | 2.92 |
|  | test | -2.300 | 1 | -1.95 | inactive | 2.98 |
|  | test | -2.300 | 1 | -2.05 | inactive | 2.92 |
|  | training | -2.300 | 1 | -2.20 | inactive | 2.92 |
|  | training | -2.000 | 1 | -1.95 | active | 2.91 |
|  | training | -1.880 | 1 | -1.70 | active | 2.99 |
|  | training | -2.000 | 1 | -2.05 | active | 2.91 |
|  | training | -2.440 | 1 | -2.41 | inactive | 2.91 |
|  | test | -2.600 | 1 | -2.05 | inactive | 2.95 |

**Supplementary Table 3.** Score of different parameters of the pharmacophoric hypothesis and PLS statistical parameters and 3D-QSAR model.

| Survival | Survival inactive | Post-doc | Site | Vector | Volume | Selectivity | #Matches |
| --- | --- | --- | --- | --- | --- | --- | --- |
| 3.93 | 1.01 | 3.93 | 1.00 | 1.00 | 0.93 | 0.94 | 8 |

| Factors | SD | R^2^ | F | P | Stability | RMSE |
| --- | --- | --- | --- | --- | --- | --- |
| 1 | 0.08 | 0.88 | 70.3 | 1.51 | 0.87 | 0.46 |

**Supplementary Figure 1.** Planktonic cell growth and extracellular indole production by *V. parahaemolyticus* in mLB broth at 30ºC with 250 rpm.

**Supplementary Figure 2.** Effects of indole and halogenated indoles on biofilm formation and planktonic cell growth against *V. harveyi*. The antibiofilm activity of 16 halogenated indoles and indole **(A)**. Antibiofilm activity of four selected halogenated indoles and indole **(B)**.
